# The evidence for old age motor neuron death: A scoping review

**DOI:** 10.14814/phy2.70967

**Published:** 2026-06-05

**Authors:** Sang Won Cheung, Matthew J. Fogarty

**Affiliations:** ^1^ Department of Physiology & Biomedical Engineering Mayo Clinic Rochester Minnesota USA; ^2^ Department of Neurology Mayo Clinic Rochester Minnesota USA

**Keywords:** anterior horn, brainstem, cervical, facial, hypoglossal, lumbar, nucleus ambiguus, phrenic, spinal cord, ventral horn

## Abstract

Age‐related neuromuscular dysfunction is widely recognized; however, the extent to which aging is accompanied by motor neuron (MN) loss remains underexplored. This review evaluates evidence for MN loss with aging across species and anatomical motor pools. We synthesize PUBMED studies in humans, non‐human primates, rats and mice within the spinal cord and brainstem. Attention was given to the methodological approach used to quantify MNs and categorized based on what MNs were quantified (specific pools, vertebral levels, lateral motor columns). Methods were assessed qualitatively, based on stereological and non‐stereological density counting approaches, and the staining techniques identified as optimal or suboptimal by defined criteria. To facilitate cross‐study comparison we conducted quantitative assessments including: coefficients of variance, percentage differences between groups and standardized effect sizes calculated with Cohen's *d*. Across species and anatomical regions, the overall literature supports the presence of age‐related MN loss. Evidence is strongest in studies using robust counting approaches such as stereology, and appropriate MN markers. As age‐related MN degeneration is more heavily investigated and becomes a locus of intervention, future work must employ rigorous methodological standards to accurately determine the extent, regional specificity, and functional consequence of aging MN loss.

## Graphical abstract

We systematically evaluated the extant literature on age‐related motor neuron (MN) death in aging, focusing on studies directly assessing MNs. We show that in the spinal cord and brainstem, there is robust evidence for MN loss, particularly in the human spinal cord. In rats, MN loss was readily observed across a variety of labelling methods and regions, while in mice, observations of MN death were equivocal.

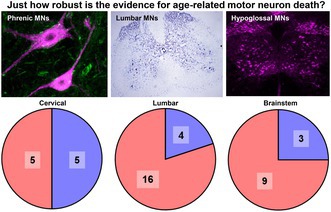

## 1 INTRODUCTION

Motor neurons (MNs) are central to all voluntary and reflex movement. Effective motor behaviors can be considered to be the correct sum of input side divers (including contextual cues, pattern selection and generation, arousal) with output fidelity (including orderly motor unit recruitment/activation, integrity of neuromuscular junctions [NMJs], fatigue) (Gandevia, 1999; Heckman & Enoka, 2012; Hudson et al., 2019; Manuel et al., 2019; Powers & Heckman, 2017). A major limit on motor performance is the amount of MNs remaining in a particular pool, which is often meticulously and rigorously evaluated in Amyotrophic Lateral Sclerosis (ALS) patients and rodent models (Fogarty et al., 2024; Kiernan & Hudson, 1991; Lee et al., 2013; Messi et al., 2007; Steyn et al., 2013). A reduced number of MNs effectively lowers the ceiling of force/torque/pressure generation of a particular muscle. This reduced capacity may be dependent or independent of muscle fiber contractility (Cheng et al., 2019; Fogarty, 2019; Miyata et al., 1995).

Surprisingly, despite the obvious decline in motor function with age, MN number in aging has not received much attention in preclinical models and human studies, beyond some hyped findings (Maxwell et al., 2018). These dysfunctions range from the mild motor impairments of the general aging population to the more severe syndrome of sarcopenia (Fogarty, 2025a; Ingram et al., 2023; Larsson et al., 2019; Lord et al., 2016). Mild motor impairment is primarily influenced by network and synaptic factors (Castro et al., 2023; Christensen & Fogarty, 2026; Fogarty, 2025a, 2025b; Maxwell et al., 2018), however, overt weakness and sarcopenia likely has major contributions from MN death and striated muscle denervation (Delbono, 2003; Doherty et al., 1993; Hepple & Rice, 2016; Larsson et al., 2019; Willadt et al., 2018). In this review we provide a critical appraisal of the methodology and results, calculating effect sizes (see (Gandevia, 2021; Gandevia et al., 2021)), to evaluate MN number in aging humans and a variety of model species. Using a critical framework of best practise for MN counting (Ferrucci et al., 2018), we synthesize these results in the context of overall MN numbers, the number of MNs within the entirety of a particular vertebral level, MNs in discrete anatomical regions (e.g., brainstem versus lumbar motor pools) and in specific retrograde labeled motor pools. These divisions are not purely academic pedantry, as modern quantification approaches in brain regions such as the hippocampus show marked differences across subregions in human neuronal loss (Fu et al., 2015). We conclude by outlining the relationship of human age‐related MN loss with that of different preclinical aging models. We integrate these insights within the framework of gerontological motor decline and muscle weakness. We also outline our working hypothesis of selective motor unit vulnerability and highlight key outstanding questions on MN neurobiology in aging.

## 2 METHODS

### 2.1 Search terms, definitions & scope

We used combinations of the following terms in PUBMED to yield the quantitative original studies evaluated in our review. For MNs: “motoneuron” and “motor neuron” alongside “anterior horn” or “ventral horn” to capture older studies. For aging: “aging,” “aging,” “aged,” “age‐related” and “old.” We selected studies that were extant in our Institutional library (hardcopy or electronic) or available online. We excluded all species except for humans, non‐human primates and rodents (rats and mice). Except where dual direct MN number assessments were performed to corroborate motor unit number estimation (MUNE) evaluations, we ignored papers with uncorroborated axonal or MUNE efforts, as many nerves comprise mixed/motor and sensory fibers and declining NMJ function is a major confounder of MUNE evaluation. Studies with no “young” control group were excluded, such as otherwise relevant studies focused on a specific age‐associated neurodegenerative condition.

**FIGURE 1:**
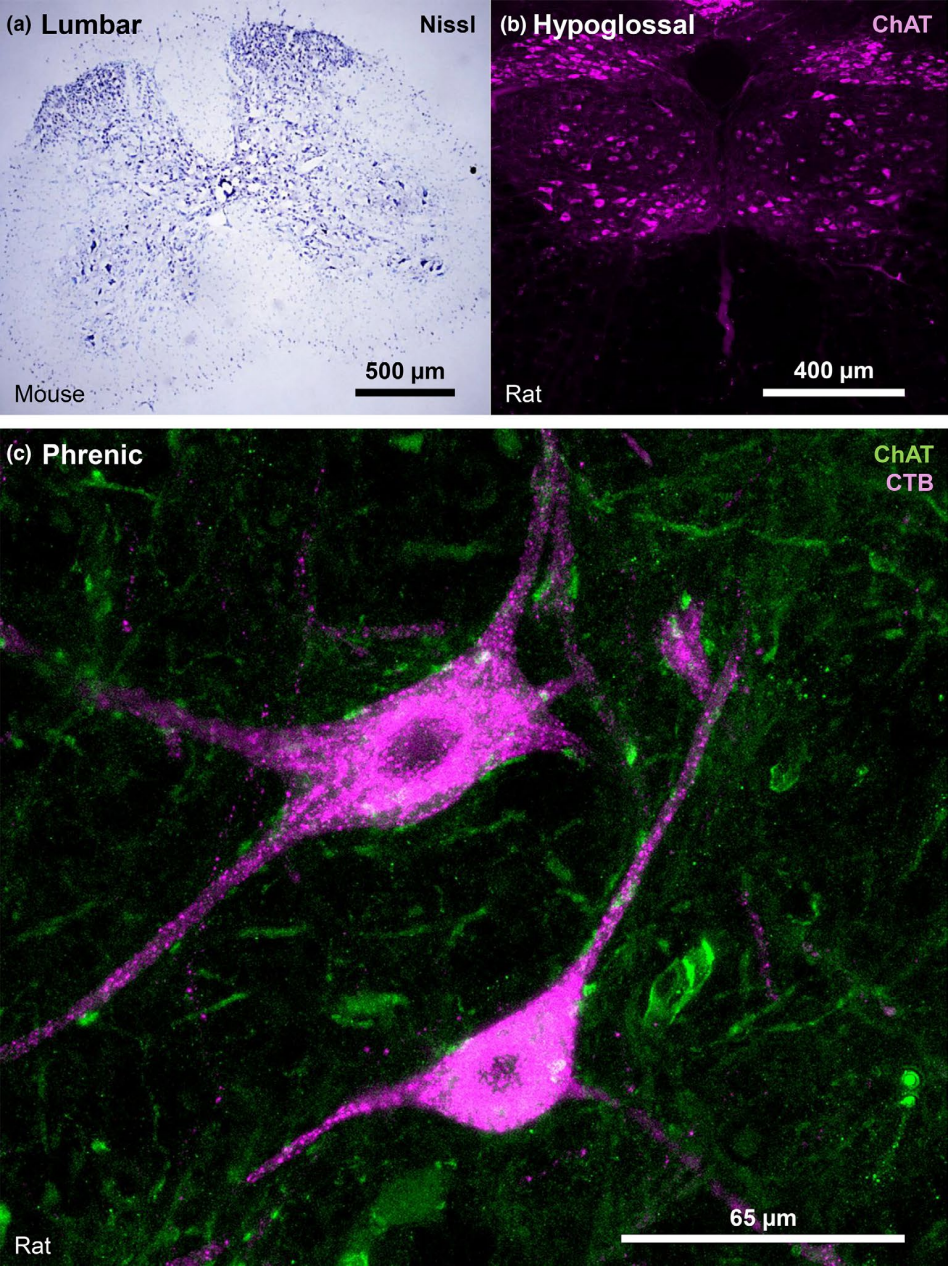
Techniques for identifying motor neurons. (a) Pictomicrograph of a mouse lumbar spinal cord section (16 μm) stained with Nissl, showing large limb innervating MNs in the ventral horn and lateral motor column. (b) Confocal z‐stack of the rat medulla (40 μm), showing the hypoglossal motor nucleus, with hypoglossal MNs immuno‐labeled with ChAT (purple). (c) Confocal *z*‐stack of the rat cervical spinal cord (75 μm), showing phrenic MNs retrogradely labeled with Alexa488‐conjugated cholera toxin β (CTB, purple) and co‐immuno\‐labeled with ChAT (green).

“Generalized” MN counts refer to approaches where the method does not distinguish a particular spinal region and/or exclusive counting of the lateral motor column or a particular brainstem motor nucleus. “Anatomical” MN counts refer to MN assessments in discrete brainstem pools or in the lateral motor columns. “Specific” MN pool evaluations are reserved for retrograde approaches. When the original author's interpreted anatomical‐based isolation of specific regions to be sufficiently definitive for a particular pool, we disagree and place these data under the classification of “anatomical” MNs. “Stereological” approaches are defined as sampling the entire region of interest with a systematically random approach, consistent with best practise (Ferrucci et al., 2018; Slomianka, 2020). “Non‐stereological” approaches either fail to sample the entirety of a region, use a mix of section orientations and/or provide a density estimate. An assortment of approaches are used to identify and count MNs in the brainstem and spinal cord (Figure 1). We have chosen to divide staining approaches consistent with a stereological approach such as Nissl staining, Chat labelling or nerve‐stump retrograde approaches as “optimal,” while classifying NeuN, and HRP and other intramuscular labelling approaches as “sub‐optimal.” The limitations of the sub‐optimal approaches relate to the capriciousness of Golgi intracellular uptake (Pilati et al., 2008), the variability and downregulation of NeuN expression in a variety of conditions (Moon et al., 2026), and the difficulty of full penetrance of intramuscular dye in muscle (Mantilla et al., 2009). Specific strains and whether an intermediate age and females and males were assessed is also noted.

**FIGURE 2:**
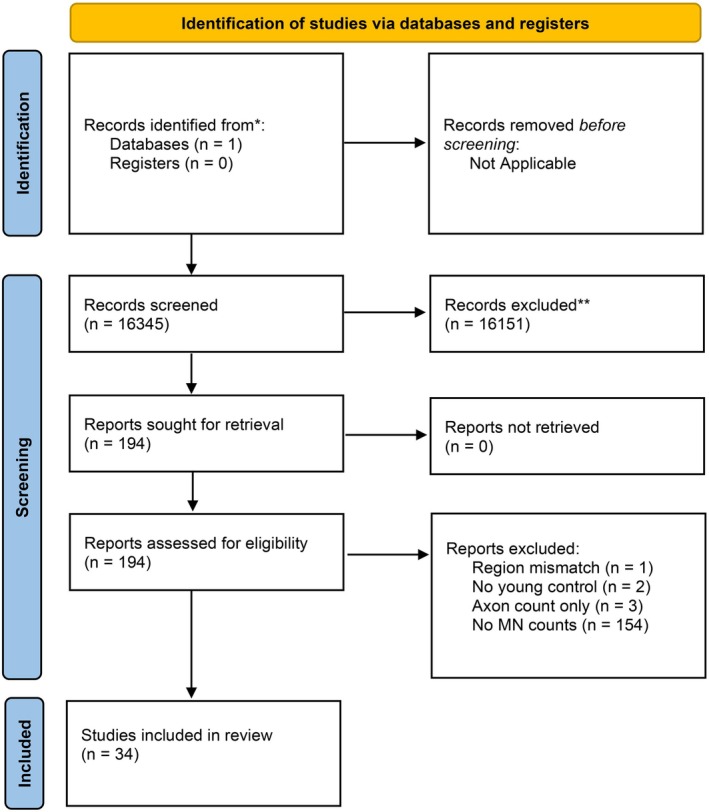
PRISMA Flowchart. Outline of NCBI Pubmed database search progression. *PUBMED database, **manually excluded based on article data.

Three quantitative evaluations performed on the data from each included study were as follows: (i) the coefficient of variance of the control (young group); (ii) the observed difference between young and old, calculated as a % of the young value; and (iii) we calculated the effect size (Cohen's *d*). Only complete data sets were considered (*n* ≥3 animals), with the oldest complete age considered “old”. We also excluded studies where no absolute MN numbers were reported. Our search process is documented in accordance with PRISMA guidelines (Figure 2 and Data S1).

## 3 RESULTS

### 3.1 Spinal MN numbers in old age

**FIGURE 3:**
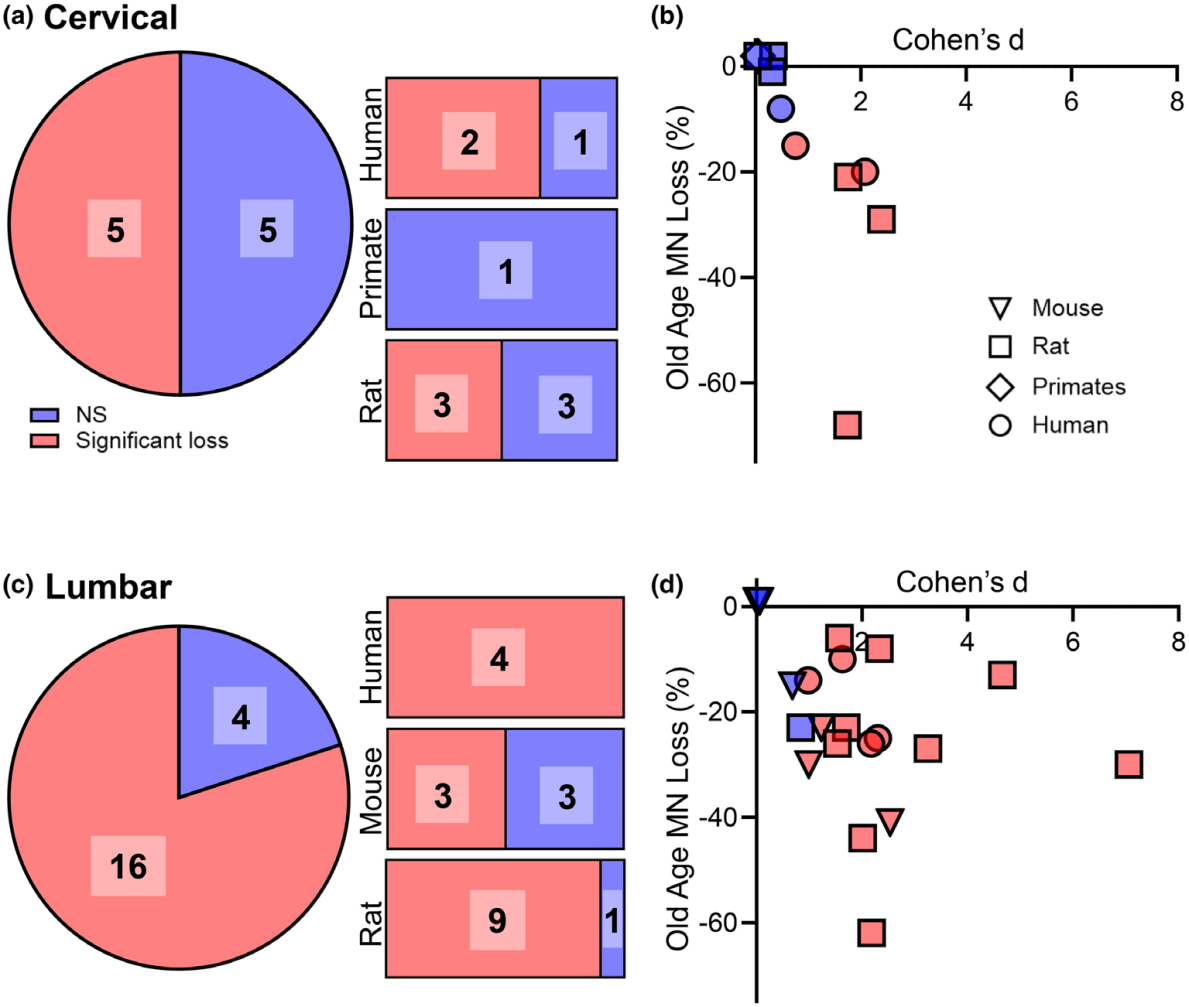
Summary of findings in studies of age‐related spinal cord MN number. (a) In cervical spinal cord, the pie chart shows the number of studies (50% of the total) showing significant MN loss in old age. The bar charts show the breakdown for specific species. (b) XY plot showing the Cohen's *d* for each study plotted against the % change from young in cervical MN studies in multiple species. (c) In lumbar spinal cord, the pie chart shows the number of studies (79% of the total) showing significant MN loss in old age. The bar charts show the breakdown for specific species. (d) XY plot showing the Cohen's *d* for each study plotted against the % change from young in lumbar MN studies in multiple species. In all graphs, blue indicated the original study's result was non‐significant, while red indicated young versus old comparison testing of *p* < 0.05. The number indicates the number of studies in each non‐significant/significant group.

Age‐related limb muscle sarcopenia and decline in motor function, manifesting as reduced strength and impaired mobility, is well documented and related to spinal MN degeneration in old age (Larsson et al., 2019). Pronounced motor deficits are associated with MN loss, axonal degeneration, and skeletal muscle degeneration (Larsson et al., 2019; Willadt et al., 2018). Beyond this, some of the earliest quantitative neuromuscular transmission failures in old age were identified in non‐limb diaphragm muscle (Kelly & Roberts, 1977), innervated by cervical phrenic MNs. Here we comprehensively assessed studies on the cervical (Table 1) and lumbar spinal cord MNs (Table 2; Figure 3). These age‐associated changes contribute to reduced force production and impaired coordination, increasing the risk of fall‐related and impaired cough‐related morbidity and mortality in the elderly (Landi et al., 2012; Tolep et al., 1995).

**TABLE 1:** Cervical MN assessments.

Species	Strain/Age/Sex	Assessment	Approach	Young CV	Δ age	Effect size
**Human**						
Zhang et al. (1996)	NA; 42–98 years^a^; FM	Anatomical, vertebral	Non‐stereological and optimal	20%	*−15%	*d* = 0.76 (medium)
Cruz‐Sanchez et al. (1998)	NA; 21–96 years^a^; FM	Anatomical, vertebral	Non‐stereological and optimal	11%	*−20%	*d* = 2.08 (large)
	young: ≤ 55 years; old: ≥ 75^a^					
Castro et al. (2023)	NA; 23–76 years^a^; MF	Anatomical, vertebral	Non‐stereological and sub‐optimal	17%	−8%	*d* = 0.48 (small)
**Primate**						
Castro et al. (2023)	NA; 8–28 years;?	Anatomical, vertebral	Non‐stereological and sub‐optimal	33%	2%	*d* = −0.05%
**Rats**						
Das et al. (2016)	SD; 150–550 days; FM	Anatomical, vertebral	Stereological and optimal	15%	*−68%	*d* = 1.75 (large)
*PHRENIC*						
**Rats**						
Fogarty et al. (2018)	F344; 6 and 24 months; FM	Specific	Stereological and optimal	9%	*−21%	*d* = 1.75 (large)
Fogarty & Sieck (2023)	F344; 6 and 24 months; FM	Specific	Stereological and sub‐optimal	11%	*−29%	*d* = 2.39 (large)
*ULNAR*						
**Rats**						
Hashizume & Kanda (1990)	F344/DuCrj; 9 and 27; M	Specific	Sterological and optimal	6%	2%	*d* = 0.34 (small)
Hashizume & Kanda (1995)	F344; 9 and 27; M	Specific	Stereological and sub‐optimal	2%	−1%	*d* = 0.32 (small)
*TRICEPS BRACHII*						
**Rats**						
Pannerec et al. (2016)	Wistar; 9, 23 months; M	Specific	Non‐stereological and sub‐optimal	23%	2%	*d* = −0.04 (none)

*Note*: Δ age denotes change between young and old, expressed as a % change of young value. ?, not disclosed in study.

^a^ young: < 55 years; old: > 75 years.

* Denotes *p* < 0.05 between ages.

**TABLE 2:** Lumbar MN assessments.

Species	Strain/Age/Sex	Assessment	Approach	Young CV	Δ age	Effect size
**Human**						
Cruz‐Sanchez et al. (1998)	NA; 21–96 years^a^; FM	Anatomical, vertebral	Non‐stereological and optimal	12%	*−25%	*d* = 2.3 (large)
Kawamura et al. (1977)	NA; 17–82 years^a^; M	Anatomical, vertebral	Non‐stereological and optimal	15%	*−14%	*d* = 0.98 (large)
Terao et al. (1996)	NA; 18–100 years^a^; MF	Anatomical, vertebral	Non‐stereological and optimal	7%	*−10%	*d* = 1.63 (large)
Tomlinson & Irving (1977)	NA; 13–95 years^a^; MF	Anatomical, vertebral	Stereological and optimal	7%	*−26%	*d* = 2.17 (large)
**Mice**						
Blasco et al. (2020)	C57BL/6J; 2, 7, 14, 27 months; M	Anatomical, vertebral	Stereological and optimal	18%	−15%	*d* = 0.68 (medium)
Chai et al. (2011)	C57BL/6J; 3, 29 months; F	Anatomical, LMC	Non‐stereological and optimal	23%	1%	*d* = −0.01 (none)
Gillon et al. (2018)	C57BL/6J; 6, 22 months; F	Anatomical, LMC	Stereological and optimal	22%	*−30%	*d* = 0.99 (large)
Piekarz et al. (2020)	C57BL/6J; 5, 17, 26 months; MF	Anatomical, vertebral	Non‐stereological and sub‐optimal	22%	*−41%	*d* = 2.53 (large)
Piekarz et al. (2022)	C57BL/6J; 5, 25 months; MF	Anatomical, vertebral	Non‐stereological and sub‐optimal	22%	*−23%	*d* = 1.23 (large)
Castro et al. (2023)	C57BL/6J; 3, 23 months; MF	Anatomical, vertebral	Non‐stereological and optimal	24%	1%	*d* = −0.06 (none)
**Rats**						
Chopek & Gardiner (2010)	F344/BN; 8, 31 months; F	Anatomical, LMC	Stereological and optimal	33%	−23%	*d* = 0.84 (large)
Jacob (1998)	F344; 6, 22 months; M	Anatomical, LMC	Stereological and optimal	28%	*−44%	*d* = −2.01 (large)
Rowan et al. (2012)	F344/BN; 8, 36 months; M	Anatomical, LMC	Stereological and optimal	13%	*−27%	*d* = −3.25 (large)
***Medial gastrocnemius***						
**Rats**						
Hashizume et al. (1988)	F344/DuCrj; 5, 12, 26, 31 months; M	Specific	Stereological and sub‐optimal	2%	*−30%	*d* = 7.06 (large)
Hashizume & Kanda (1990)	F344/DuCrj; 9, 27 months; M	Specific	Stereological and optimal	5%	*−6%	*d* = 1.57 (large)
Hashizume & Kanda (1995)	F344; 9 and 27 months; M	Specific	Stereological and sub‐optimal	4%	*−8%	*d* = 2.33 (large)
Kanda (2002)	F344/DuCrj; 16, 28 months; M	Specific	Stereological and sub‐optimal	3%	*−13%	*d* = 4.66 (large)
Pannerec et al. (2016)	Wistar; 9, 19, 23 months; M	Specific	Non‐stereological and sub‐optimal	37%	*−62%	*d* = 2.18 (large)
*Tibialis anterior*						
**Rats**						
Ishihara et al. (1987)	Wister albino; 10, 65, 135 weeks; F	Specific	Stereological and sub‐optimal	17%	*−23%	*d* = 1.7 (large)
*Soleus*						
**Rat**						
Ishihara et al. (1987)	Wister albino; 10, 65, 135 weeks; F	Specific	Stereological and sub‐optimal	21%	*−26%	*d* = 1.53 (large)

*Note*: Δ age denotes change between young and old, expressed as a % change of young value.

^a^ young: < 55 years; old: > 75 years.

* Denotes *p* < 0.05 between ages.

#### 3.1.1 Cervical MNs

The cervical MNs are organized into longitudinal motor columns spanning the rostrocaudal axis of the spinal cord, exhibiting somatotopy such that MNs in the ventromedial column preferentially innervate axial trunk muscles, and those of the lateral column preferentially innervate the shoulder, and proximal and distal muscles of the upper/forelimbs. The lateral column also exhibits rostrocaudal and dorsoventral somatotopy such that the shoulder muscles are preferentially innervated by rostral and ventral MNs, whilst the upper/forelimb muscles are preferentially innervated by caudal and dorsal MNs (Bacskai et al., 2013; Callister et al., 1987; McHanwell & Watson, 2009; McKenna et al., 2000; Richmond et al., 1978; Romanes, 1941; Sengul et al., 2012; Shinoda et al., 1997; Sterling & Kuypers, 1976; Tosolini & Morris, 2012; Watson et al., 2009). The ventromedial MNs innervate the axial trunk muscles (biventer cervicis, splenius, complexus, longissimus and semispinalis cervicis, and longissimus capitis) (Callister et al., 1987; Fritz et al., 1986; Richmond et al., 1978; Shinoda et al., 1997; Veshchitskii et al., 2025a). The rostral (C2‐C6) and ventrolateral MNs innervate the shoulder muscles (acromiotrapezius, levator claviculae, acromiodeltoideus and spinodeltoideus) (Bacskai et al., 2013; McHanwell & Watson, 2009; McKenna et al., 2000; Romanes, 1941; Sengul et al., 2012; Sterling & Kuypers, 1976; Tosolini & Morris, 2012; Watson et al., 2009). The intermediate rostrocaudal (C3‐C8) and mid‐lateral MNs innervate the proximal upper/forelimb muscles (triceps brachii and biceps brachii) (Bacskai et al., 2013; McHanwell & Watson, 2009; McKenna et al., 2000; Romanes, 1941; Sengul et al., 2012; Sterling & Kuypers, 1976; Tosolini & Morris, 2012; Watson et al., 2009). The caudal (C4‐T1) and dorsolateral MNs innervate the distal upper/forelimb muscles (flexor digitorum profundus, flexor capri radialis, palmaris longus extensor carpi radialis, and pronator teres) (Bacskai et al., 2013; McHanwell & Watson, 2009; McKenna et al., 2000; Romanes, 1941; Sengul et al., 2012; Sterling & Kuypers, 1976; Tosolini & Morris, 2012; Watson et al., 2009). Prior tracing studies show that these MN pools exhibit substantial overlap along the rostrocaudal and ventrolateral axis (McKenna et al., 2000; Tosolini & Morris, 2012). The diaphragm muscle is innervated by phrenic MNs (Figure 1) residing in central to caudal (C3‐C5, with some species variation shifting more caudal) shifting in the gray matter from the ventrolateral portion more rostrally to the ventromedial portion more caudally (Duron et al., 1979; Goshgarian & Rafols, 1981; Keswani & Hollinshead, 1955; Kuzuhara & Chou, 1980; Qiu et al., 2010; Sterling & Kuypers, 1976; Takahashi et al., 1980; Webber et al., 1979).

In humans, all two studies with reasonable numbers of aged individuals (10–12) reported modest MN loss (~15–20%, with medium and large Cohen's *d*) at cervical vertebral levels (Cruz‐Sanchez et al., 1998; Zhang et al., 1996), with the negative study having only three subjects per age and MN density counted in a total of two 50 μm sections (Castro et al., 2023) (Table 1). The latter study evaluated cervical MN density in non‐human primates with similarly lesser rigor (Castro et al., 2023). Given the diversity apparent in human aging, we consider it wise to power for greater variability on human studies compared to those of in‐ (and even out‐) bred rodents, with *n* = 3 insufficient even in the most optimistic rodent study.

In rats, cervical MN loss across the lateral motor column has been evaluated in 100‐ versus 500‐days‐old Sprague Dawley rats, with a massive decline in MN number (~70%, large Cohen's *d*) (Das et al., 2016) (Table 1). Despite this, in F344 rats where individual motor pools were assessed, no changes were observed in old age ulnaris, via nerve‐dip approaches (Hashizume & Kanda, 1990, 1995) or triceps via intramuscular approaches (Pannerec et al., 2016) (Table 1). By contrast, in phrenic MNs, substantial (20%–30%, large Cohen's *d*) age‐related losses are observed in both nerve‐dip techniques (Fogarty et al., 2018) and in approaches utilizing NMJ retrograde transport (Fogarty & Sieck, 2023) (Table 1). We were unable to uncover any studies looking at cervical MNs across the lifespan in mice of any strain.

Overall, two of the three human studies showed modest cervical MN loss, fitting with the motor pool dependent findings in rats (Figure 3). The marked decrease in lateral motor column numbers is likely decreased in approaches where all MNs in a vertebral level are assessed, due to the resilience of the postural axial muscles to degeneration. Assessing total lateral motor column MN number in the entirety of the cervical cord of human, non‐human primate, and other model species would go a long way to clarifying this issue.

#### 3.1.2 Lumbar MNs

The lumbar MNs are organized into motor columns in the ventral horn (Figure 1), exhibiting somatotopy such that MNs in the ventromedial column preferentially innervate axial and proximal lower/hindlimb muscles, and those of the ventrolateral column preferentially innervate distal muscles of the lower/hindlimbs (Bacskai et al., 2014; Ferrucci et al., 2018; Fulceri et al., 2012; McHanwell & Watson, 2009; Sengul et al., 2012; Watson et al., 2009). The ventromedial MNs innervate the axial and proximal lower/hindlimb muscles (interspinales lumborum, quadratus lumborum, multifidus, flexor caudae, psoas minor and psoas major, and gracilis anterior) (Bacskai et al., 2014; McHanwell & Watson, 2009; Takahashi et al., 2010; Veshchitskii et al., 2025b). The ventrolateral MNs innervate the distal lower/hindlimb muscles (gastrocnemius, peroneus longus/brevis, and tibialis anterior) (Bacskai et al., 2014; McHanwell & Watson, 2009; Qi et al., 2022). Although the overall organization is medial‐axial and lateral‐distal, specific mapping studies demonstrate a partial overlap between the ventromedial and ventrolateral MNs, with some axial and proximal hindlimb muscles (quadratus lumborum, flexor caudae, psoas minor and major, rectus abdominis, iliacus and gluteus medius) innervated by ventrolateral MNs and some distal hindlimb muscles (gastrocnemius, soleus, peroneus longus/brevis, vastus lateralis, and semitendinosus) innervated by ventromedial MNs (Bacskai et al., 2014; McHanwell & Watson, 2009; Takahashi et al., 2010; Veshchitskii et al., 2025b).

In humans, all studies report similar results of reductions in old lumbar MN numbers ranging from ~10% to 26% loss (with a large Cohen's *d*) (Cruz‐Sanchez et al., 1998; Kawamura et al., 1977; Terao et al., 1996; Tomlinson & Irving, 1977). Notably, only one study utilized the stereological approach, showing the greatest reduction in old lumbar MNs (~26%) (Tomlinson & Irving, 1977) (Table 2).

In rats, anatomical assessment of the lateral motor column in F344/BN and F344 rats shows reductions in old lumbar MN numbers (~30%–45%), with a large Cohen's *d* (Chopek & Gardiner, 2010; Jacob, 1998; Rowan et al., 2012) (Table 2). Importantly, only the F344 study which included male rats was statistically significant (Jacob, 1998), whereas the F344/BN study which used female rats was not statistically significant despite a larger sample size (Chopek & Gardiner, 2010), with the third F344/BN male study showing a significant difference (Rowan et al., 2012). The female rats in the “old” group were also 7 months younger than the “old” male group in the F344/BN studies (Chopek & Gardiner, 2010; Rowan et al., 2012). Studies in rats have also investigated specific motor nuclei within the lumbar spinal cord using retrograde labelling approaches. In the gastrocnemius motor nucleus, statistically significant reductions are observed in old male F344/DuCrj rats (~6%, ~13% and ~30%) (Hashizume et al., 1988; Hashizume & Kanda, 1990; Kanda, 2002), male F344 rats (~8%) (Hashizume & Kanda, 1995) and male Wistar rats (~62%) (Pannerec et al., 2016), all with a large Cohen's *d* (Table 2). These gastrocnemius results are confusing as the same group reports a wide range of overall differences (6%–30%) using an identical approach (Hashizume et al., 1988; Hashizume & Kanda, 1990; Kanda, 2002). Specific labelling of the tibialis anterior and soleus muscle in Wister albino rats both show a reduction in old retrogradely‐labeled lumbar MNs (~23% and ~26% respectively with a large Cohen's *d*); however, only the reductions in old MNs innervating the tibialis anterior were statistically significant (Ishihara et al., 1987) (Table 2).

In mice, anatomical assessment of the lateral motor column in C57BL/6J mice shows contrasting results, with reductions (~30%, large Cohen's *d*) (Gillon et al., 2018) and trivial increases in old lumbar MN numbers (~1%, no effect size) both being observed (Chai et al., 2011) (Table 2). Important to note, only Gillon et al. (2018) used a stereological approach. Anatomical assessment of the entire lumbar ventral horn also demonstrates mixed results, ranging from trivial increases (~1%, no effect size) (Castro et al., 2023) and statistically insignificant reductions using stereological and optimal approaches (~15%, medium Cohen's *d*) (Blasco et al., 2020) and non‐stereological and sub‐optimal approaches (~23%, large Cohen's *d*) (Piekarz et al., 2022) to statistically significant reductions using non‐stereological and sub‐optimal approaches (~41%, large Cohen's *d*) (Piekarz et al., 2020) (Table 2).

Overall, all human studies assessed showed a significant decrease in lumbar MNs (~10–25%), with a large Cohen's *d*, with is likely an underestimate due to the inclusion of the relatively resilient axial motor pools (Figure 3). Rat data was equivocal as far as study significance (likely due to the large co‐efficient of variation) when looking at lateral motor column MN numbers, although the negative study was performed in a strain (F344/BN) without noticeable MN loss in other studies. In rats, females may be more muted in MN loss at the same age, as they do have a slightly longer survival curve than males (i.e., they age slightly slower), thus a 36‐ month‐old male may be more ravaged by neurodegeneration than an identically aged females (Turturro et al., 1999). Individual medial gastrocnemius, tibialis anterior and soleus motor pools were robustly affected, with only a single study showing loss of under 10%. Similarly, mouse studies were also equivocal, with the vast majority non‐stereological and using sub‐optimal preparations. It is tempting to assume that bad habits (namely section density counts on a minimal stereological selection of sections) from the severely affected ALS mouse models have been used as templates for aging assessments in mice.

### 3.2 Brainstem MN numbers in old age

**FIGURE 4:**
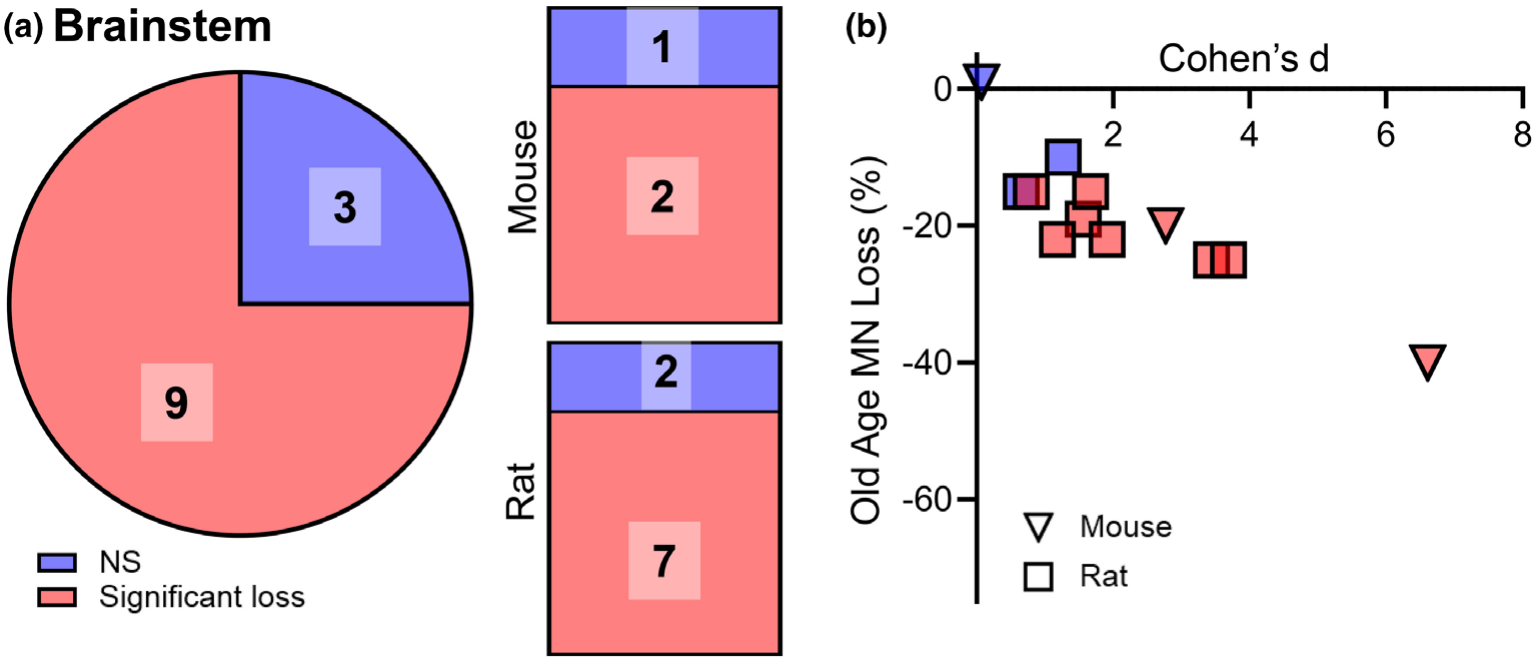
Summary of findings in studies of age‐related brainstem MN number. (a) In brainstem, the pie chart shows the number of studies (73% of the total) showing significant MN loss in old age. The bar charts show the breakdown for specific species. (b) XY plot showing the Cohen's *d* for each study plotted against the % change from young in brainstem MN studies in multiple species. In all graphs, blue indicated the original study's result was non‐significant, while red indicated young versus old comparison testing of *p* < 0.05. The number indicates the number of studies in each non‐significant/significant group.

A great many brainstem‐related motor behaviors such as swallow, speech, coughing and the maintenance of airway patency during sleep are impaired in old age (Baum & Bodner, 1983; Crow & Ship, 1996; Enright et al., 1994; Gaspar et al., 2017; Marino & Johns 3rd, 2014; Thiyagalingam et al., 2021; Turley & Cohen, 2009; Youmans et al., 2009). Although subtle age‐associated changes centre on circuit and signal integration disruptions (Fogarty, 2025a), more severe deficits involve weakness and denervation of the musculature (Crow & Ship, 1996; Hodges et al., 2004; Kemp et al., 2026; Larsson et al., 2019; Sieck et al., 2023). Notably, deficits in airway protective behaviors (eg. weak cough and dysphagia) predispose to age‐related aspiration pneumonia, a major cause of morbidity and mortality in the elderly (Ebihara et al., 2012; Janssens & Krause, 2004; Langmore et al., 1998; Martin et al., 1994; Nativ‐Zeltzer et al., 2022). Here we comprehensively assessed studies on the facial, hypoglossal and nucleus ambiguus motor nuclei MNs (Table 3; Figure 4).

**TABLE 3:** Brainstem MN assessments.

Species	Strain/Age/Sex	Assessment	Approach	Young CV	Δ age	Effect size
*Facial*						
**Rat**						
Johnson & Duberley (1998)	Wistar; 6 and 25 months; M	Anatomical	Stereological and optimal	11%	*−22%	*d* = 1.91 (large)
Johnson & Duberley (1998)	F344; 6 and 25 months; M	Anatomical	Stereological and optimal	10%	−10%	*d* = 1.27 (large)
Aperghis et al. (2003)	SD; 6 and 24 months; M	Anatomical	Stereological and optimal	14%	*−15%	*d* = 0.76 (medium)
Katharesan et al. (2016)	SD; 3 and 24 months; F	Anatomical	Stereological and optimal	13%	*−22%	*d* = 1.18 (large)
**Mice**						
Sturrock (1988)	ASH/TO; 6, 21, 25, 28 and 31;?	Anatomical	Stereological and optimal	9%	*−20%	*d* = 2.77 (large)
*Hypoglossal*						
**Rat**						
Fogarty (2023)	F344; 6 and 24 months; FM	Anatomical	Stereological and optimal	11%	*−15%	*d* = 1.67 (large)
Schwarz et al. (2009)	FBN; 10, 24 and 32 months; M	Anatomical	Non‐stereological and sub‐optimal	20%	−15%	*d* = 0.68 (medium)
Fogarty et al. (2026)	F344; 6, 18 and 24 months; FM	Anatomical	Stereological and optimal	10%	*−25%	*d* = 3.69 (large)
**Mice**						
Sturrock (1991)	?; 6, 25 and 28 months;?	Anatomical (Varied)	Non‐stereological and optimal	8%	1%	*d* = 0.07 (none)
*Nucleus Ambiguus*						
**Rat**						
Basken et al. (2012)	F344/BN; 9 and 32 months; M	Thyroarytenoid	Non‐stereological and sub‐optimal	27%	*−25%	*d* = 3.44 (large)
Fogarty (2025b)	F344; 6, 18 and 24 months; FM	Anatomical	Stereological and optimal	11%	*−19%	*d* = 1.56 (large)
**Mice**						
Sturrock (1990)	ASH/TO; 6, 21, 25, 28 and 31;?	Anatomical	Stereological and optimal	1%	*−40%	*d* = 6.61 (large)

*Note*: Δ age denotes change between young and old, expressed as a % change of young value. ?, not disclosed in study.

* Denotes *p* < 0.05 between ages.

#### 3.2.1 Facial MNs

The facial MNs innervate the many facial muscles responsible for whisking and sound location identification in rodents and for facial expression and vocalization in primates, and innervating the stylohyoid and digastric muscles, required for swallowing and chewing (Cattaneo & Pavesi, 2014; Guest et al., 2018; Hattox et al., 2002; Noohi et al., 2024; Sherwood, 2005). Thus, facial MNs, although they serve different purposes across species, are highly important for successful animal function and an important neuronal bell‐weather for evaluating the neuromotor system across species (Sherwood, 2005; Watson et al., 2012). Indeed, in humans, facial MN dysfunction contributes to motor symptoms in a variety of neurodegenerative diseases (Carswell et al., 2015; De Oliveira et al., 2022; Farrugia et al., 2006, 2007; Miyata et al., 2020), in addition to age‐related pathophysiological implications for Bell's Palsy, where recovery rates in the elderly are reduced compared to the young (Danielidis et al., 1999).

A majority of facial motor nucleus evaluations centre upon facial nerve anastomosis and axotomy studies (Bendella et al., 2018; Tetzlaff et al., 1991; Yan et al., 1995). Although no detailed evaluations of facial MN numbers are available in aging humans, a few studies have evaluated facial MN numbers in old age in a variety of rat strains (Aperghis et al., 2003; Johnson & Duberley, 1998; Katharesan et al., 2016) and in mice (Sturrock, 1988) (Table 3). In all studies, Cohen's *d* effect sizes were medium (one study) to large (the remaining four), with modest MN loss ~10%–20% in rats (Aperghis et al., 2003; Johnson & Duberley, 1998; Katharesan et al., 2016) and 20% in mice (Sturrock, 1988).

#### 3.2.2 Hypoglossal MNs

Hypoglossal MNs (Figure 1) innervate the intrinsic superior and inferior longitudinals, transversalis and verticalis muscles, as well as the extrinsic genioglossus, styloglossus, hyoglossus and geniohyoid muscles (Sawczuk & Mosier, 2001). Hypoglossal motor nucleus organization does exhibit some somatotopy, with the retractor muscles (longitudinal, hyoglossus and styloglossus) innervated by the ventral region and protrudor muscles (transversalis, verticalis, and geniohyoid) innervated by the dorsal region (Dobbins & Feldman, 1995; McClung & Goldberg, 2000; Wealing et al., 2019). The main protrudor muscle, the genioglossus, is innervated by the ventral and ventrolateral regions (Frazure et al., 2025; Schwarz et al., 2009; Wealing et al., 2019).

A relative paucity of studies directly evaluate hypoglossal MN survival in old age, with no age‐controlled evaluations in humans. In rats, different studies in F344/BN and F344 rats reported contrasting results (Fogarty, 2023; Fogarty et al., 2026; Schwarz et al., 2009), with all assessments showing ~ >5% reductions in old hypoglossal MN numbers (with medium to large Cohen's *d*); only the F344 studies were statistically significant (Table 3). Importantly, the negative F344/BN study relied on density estimates rather than on evaluating the entire nucleus (Schwarz et al., 2009), which had a slightly exaggerated loss (~20%) of ventrolateral genioglossus‐innervating MNs (Fogarty, 2023). Importantly, the latter study compared in toto and density estimates within the same samples, showing density estimates were insufficient to determine MN loss, likely due to marked regional variations in the number of MNs (Fogarty, 2023). In the F344/BN study, the age (32 months) may not have been sufficiently “old” to observe any changes, seen at 38 months in lumbar F344/BN MNs (Rowan et al., 2012). In mice, it was reported that no hypoglossal MN loss occurred in age, although this study combined transverse and parasagittal sectioning techniques (Sturrock, 1991).

#### 3.2.3 Nucleus Ambiguus MNs

The nucleus ambiguus consists of three regions, the more rostral compact, the medial semi‐loose, and the more distal loose formation, with older nomenclatures also including the retrofacial nucleus (Bieger & Hopkins, 1987; Fryscak et al., 1984; Portillo & Pasaro, 1988a, 1988b; Sturrock, 1990). The somatotopic organization of the nucleus is such that the rostral compact formation innervates the esophageal MNs, while the more caudal semicompact and loose formations innervate the palatopharyngeal and laryngeal MNs, respectively (Bieger & Hopkins, 1987; Fryscak et al., 1984; Portillo & Pasaro, 1988b).

Despite dysphagia being a major indicator of morbidity and mortality in the elderly, there are only three studies, two in rats (Basken et al., 2012; Fogarty, 2025b) and the other in mice (Sturrock, 1990) evaluating nucleus ambiguus MN survival in aging. Two of these studies used a reliable Nissl approach and evaluated the entire motor nuclei rather than density estimates (Fogarty, 2025b; Sturrock, 1990), with the other looking at a specific muscle, the thyroarytenoid via intramuscular CTB and using a skewed density estimate (5 sections with the greatest MN number per rat) (Basken et al., 2012) (Table 3). All studies exhibited large effects relative to controls and based on Cohen's *d*. It is worth knowing that one of, if not the only mouse MN pool to see reduced numbers in old age is the nucleus ambiguus, with MNs being decimated by an ~40% loss (Sturrock, 1990), almost twice that of F344 rats (Fogarty, 2025b) and F344 hybrids (Basken et al., 2012) (Table 3). Not assessed in our analysis but considered in two of these papers are intermediate late‐middle‐age group MN quantifications, showing nucleus ambiguus MN numbers are relatively stable before rapid decline from late‐middle to old age (Fogarty, 2025b; Sturrock, 1990).

## 4 DISCUSSION

### 4.1 Pathophysiological implications of MN death

#### 4.1.1 Spinal cord

In humans, there is an age‐related preferential reduction in large, myelinated motor fibers from the cervical spinal cord (Mittal & Logmani, 1987; Zhang et al., 1995). This is mirrored in the lumbar spinal cord, with a decrease in density of myelinated axons and axonal degeneration in older age groups (Jacobs & Love, 1985). These findings are consistent with reduction in human cervical and lumbar MUNE evaluations in old age (Campbell et al., 1973; Doherty & Brown, 1993; Galea, 1996; Piasecki et al., 2016) and with age‐related NMJ denervation (Willadt et al., 2018).

In rats and mice, age‐related denervation and muscle weakness and atrophy are readily evident, with many studies focusing on atrophy‐resilient compared to atrophy‐vulnerable muscles and/or fiber type‐dependent innervation differences (Arnold & Clark, 2023; Banker et al., 1983; Chai et al., 2011; Deschenes et al., 2010; Fogarty et al., 2019; Li et al., 2025; Padilla et al., 2021; Prakash & Sieck, 1998; Valdez et al., 2012). Of course, whether MN death is a consequence of NMJ denervation or vice versa is a key pathophysiological question.

#### 4.1.2 Brainstem

To our knowledge, there appears to be no readily available data on aging brainstem motor nuclei in humans. There have been some limited evaluations of hypoglossal (Tiago et al., 2005) and superior laryngeal motor axons in humans (Mortelliti et al., 1990). In under‐ compared to over‐sixties males, no differences in myelinated motor fibers were observed with age; however, only 7–14% of the nerve cross‐section was assessed in a non‐stereological manner (Tiago et al., 2005), a departure from other hypoglossal nerve assessments (McCall et al., 2020). By contrast, MUNE approaches in aging human genioglossus muscle exhibit motor unit expansion, consistent with hypoglossal MN loss, a study likely underestimating MN loss due to the MUNE being evaluated during ventilation alone (Saboisky et al., 2014). By contrast to the incomplete sampling of the human hypoglossal nerve, the in toto assessment of the recurrent laryngeal nerve shows a 30% decrease in myelinated (presumably motor) fibers, consistent with MN loss (Mortelliti et al., 1990).

In F344 rats and F344 hybrids, brainstem MN loss is concomitant with an increased incidence of partial or complete NMJ denervation and remodeling in laryngeal muscles (Connor et al., 2002; Johnson et al., 2013; Shembel et al., 2021) and tongue (Hodges et al., 2004; Johnson & Connor, 2011; Kemp et al., 2026; Kletzien et al., 2018). Overall MN loss and denervation are consistent with overt weakness and neuromuscular transmission deficits (Connor et al., 2009; Fogarty et al., 2026; Glass et al., 2021; Nagai et al., 2008; Sieck et al., 2023), and aerodigestive behavioral compromise (Fogarty, 2025b; Suzuki et al., 2002; Zhang et al., 2008) in rats, consistent with aging human dysphagia (Thiyagalingam et al., 2021). Overall, due to the life‐sustaining nature of aerodigestive behaviors, even modest MN loss in these pools may be highly deleterious in old age.

### 4.2 Motor unit type‐dependent MN death in aging

The overarching hypothesis of our work in the aging neuromotor system is that the MNs comprising fast fatigueable motor units are vulnerable to degeneration and subsequent death in old age (Fogarty, 2025a; Fogarty, Brown, & Sieck, 2020). This hypothesis is consistent with the selective effects of aging on type IIx/b muscle fibers (Larsson et al., 2019). Following experimental denervation and in conditions where MNs are lost, such as ALS, fast fatigueable motor units are selectively vulnerable (Fogarty, 2018a; Miyata et al., 1995; Nijssen et al., 2017). In ALS patients and model MNs, there appears to be disproportionate degenerative effects and loss of larger MNs (Dukkipati et al., 2018; Fogarty, Mu, et al., 2020; Kaplan et al., 2014; Kiernan & Hudson, 1991; Leroy et al., 2014; Pun et al., 2006), of which many are likely to be fast fatigueable MNs. Similar types of observations are found in aging, where larger MNs are lost and more vulnerable to degenerative processes (Christensen & Fogarty, 2026; Fogarty, 2023; Fogarty et al., 2018; Fogarty & Sieck, 2023; Jacob, 1998; Kawamura et al., 1977). Notably, much of this size‐dependent loss is obscured in the assessment of MNs in the lumbar spinal cord, as the MNs' greatest somal extent is in the parasagittal plane (Sterling & Kuypers, 1976).

We contend that particularly resilient motor pools are not resilient due to circuit factors or anatomical uniqueness (e.g., onuf's, occulomotor (Mannen et al., 1982; Nijssen et al., 2017)), but that all slow and fast fatigue resistant MNs are resilient, regardless of anatomical location. This is borne out in limb muscles such as the ulnaris and triceps brachii (Tables 1 & 2), where the MNs innervating them are not lost (Hashizume & Kanda, 1990, 1995; Pannerec et al., 2016), despite evidence of MN loss in the lateral motor columns, where these motor pools reside (Das et al., 2016). We assert, based on these limb observations, that the proportion of vulnerable fast fatigueable MNs to resilient slow and fast fatigue resistant MNs governs whether significant MN loss will occur in a particular pool, noting motor units with mixed or primarily fast fatigueable units, such as diaphragm (Fogarty, Marin Mathieu, et al., 2020; Moore et al., 2006; Stubbings et al., 2008), gastrocnemius (Burke et al., 1973; White et al., 2016; Zhan & Sieck, 1992), laryngeal (Rhee et al., 2004), tibialis anterior (Giacomello et al., 2020; Totosy de Zepetnek et al., 1992) and tongue (Fogarty & Sieck, 2021; Ota et al., 2005; Rahnert et al., 2010), exhibit MN death (Tables 1, 2 and 3).

The inclusion of brainstem, cervical, and lumbar assessments gives rise to the question as to which motor pools more generally are affected by aging. Our results suggest that MN loss occurs across all brainstem, cervical, and lumbar motor regions. Overall, there was a propensity for studies to report generalized cervical and lumbar MN loss in old age humans (6 of 7 studies), rats (3 of 4 studies); whereas in mice, generalized lumbar MN loss was equivocal (3 of 6 studies) (Tables 1 and 2; Figure 3). In spinal cord studies where specific pools are labeled, the comparison is often between motor pools comprising predominantly slow and fast fatigue resistant motor units to pools comprising mostly fast fatigueable motor units. Three such studies involved MN pools with and without aging loss in the same rat (Hashizume & Kanda, 1990, 1995; Pannerec et al., 2016), making trends about locations confounded as some hypotheses expected to show no differences. In the brainstem, MN loss was observed in 7 of 9 rat studies and 1 in 2 mouse studies, consistent with results in the spinal cord (Table 3; Figure 4). The reduced incidence of MN loss in aging mice is consistent with NMJ deficits being a prime driver of neuromotor decline in aged mice (Chai et al., 2011; Sieck & Fogarty, 2025) and in murine ALS models (Arbour et al., 2015; Martineau et al., 2018).

### 4.3 Future directions

The major questions to be answered regarding MN loss in aging are whether specific MN types are vulnerable, and what pathophysiological mechanism(s) contribute to their degeneration and death. If MN death is type‐specific, then ideally this mechanism would be selective for the MN type dependence, rather than a pan‐MN homogeneous malaise. Conversely, a resilience factor to whatever perturbations leading to MN death may be present in resilient MNs. It will be important to avoid common pitfalls when assessing these proposed mechanisms and any ameliorative interventions on MN survival.

To the first point, some attempts have been made to characterize MN type beyond size, where inappropriate section orientations (Sterling & Kuypers, 1976) and accumulation of lipofuscin (Gray & Woulfe, 2005) may confound somal size estimates. However, unlike the unambiguous muscle fiber type molecular signatures of myosin heavy chains (Schiaffino & Reggiani, 2011), the molecular signatures of fast fatigueable MNs, which seem promising in terms of ALS MN type vulnerability (Dukkipati et al., 2018; Kaplan et al., 2014; Manuel & Zytnicki, 2019; Pun et al., 2006), have not been fully characterized as far as their adherence to the Size‐Principle (Fogarty, 2018b). This may sound trivial, but it remains unknown if the expression of these molecular markers is repressed in scenarios of MN death or if MNs expressing these markers die. It remains a major unanswered question as to whether fast fatigueable MNs, assayed by size and molecular signature, are vulnerable across both limb and aerodigestive muscles.

The major proposed pathophysiological mechanisms for age‐related neurodegeneration centre around inflammation and mitochondrial dysfunction a (Franceschi et al., 2018; Franceschi & Campisi, 2014; Larsson et al., 2019; Vasto et al., 2007), all known to play key roles in MN degeneration in ALS (Dupuis et al., 2004; Goutman et al., 2022; Sasaki & Iwata, 2007). Indeed, mutations in the mitochondrial SOD1 gene are a major cause of familial ALS (Rosen et al., 1993), and lead to aggressive MN death in rodent models (Gurney et al., 1994). In studies in humans and rats, we and others show that systemic “inflammaging,” that is, increased inflammatory cytokines are present both in the serum (Bruunsgaard et al., 1999) but also in neural tissues (Fogarty et al., 2026; Katharesan et al., 2016; Scheinert et al., 2015) and striated muscle (Fogarty et al., 2026). In F344 rats, this generalized elevation in inflammatory cytokines is sufficient enough to provoke structural and functional mitochondrial changes of larger, likely fast fatigueable MNs, in late middle age and persisting to old age (Fogarty et al., 2026), where post mortem MN evaluation of mitochondrial function shows similar defects (Rygiel et al., 2014). Notably, these mitochondrial deficiencies in larger MNs may drive MN death (Ma et al., 2020; Schon & Manfredi, 2003) and occur prior to similar mitochondrial changes in fast fatigueable type IIx/b muscle fibers (Fogarty et al., 2026; Fogarty, Marin Mathieu, et al., 2020). Mitochondrial degeneration was also observed in MN dendrites, where integration of synaptic inputs occurs (Christensen & Fogarty, 2026), linking mitochondrial dysfunction with the long prodromal phase of slowly declining neuromotor function prior to overt MN death (Fogarty, 2025a; Ingram et al., 2023; Lord et al., 2016). The advantage of this pathophysiological framework is that it is selective for larger MNs, yet remains compatible with other proposed contributors to aging MN death such as lipofuscin accumulation (Gray & Woulfe, 2005), neurotrophic decline (Personius & Parker, 2013) and dysregulated proteostasis (Hipp et al., 2019).

When evaluating MN death, the major experimental design problems to be avoided are between subject variability and within subject variability (Slomianka, 2020). Ferruci and colleagues provide an excellent review showing these principles applied to MN number estimation (Ferrucci et al., 2018). In brief, the first can be alleviated by adequately powering a study, which usually requires relatively modest amounts of inbred or outbred rats, based on the estimated variability of young subjects. Of course, in human studies, the variability is likely to be both increased and out of the control of the investigator and must be factored into the design/enrollment. If aged subjects are limited, there are ways to account for this by increasing the “young” group size in an a priori manner. By contrast, within subject variability is entirely within the control of the investigator, and is reduced by adequate within sample assessments, ideally in a systematic random sampling stereological paradigm (Slomianka, 2020). As a rule of thumb, ~150–200 individual MN “counts” are usually sufficient to minimize within sample variability when using a stereological approach (Knudsen et al., 2010; Slomianka, 2020). Due to the variability within a motor pool, per section density estimates are usually insufficient to account for anatomical changes within the structure (Ferrucci et al., 2018; Slomianka, 2020). For example, 5 individual sample sections would require >30 MNs per section to be considered borderline sufficient as far as a sample “count”, and may not adequately sample the anatomy, particularly in regions that vary highly (Ferrucci et al., 2018; Fogarty, 2023, 2024; Slomianka, 2020). It is important to note that in the spinal cord, MN sizes will not be definitive in transverse sections and any size‐dependence (or lack of it) should be interpreted with caution (Sterling & Kuypers, 1976). In cases where intramuscular labelling is done to retrogradely label specific MNs, the integrity of the NMJ must be used to couch interpretation of the magnitude of MN loss, with nerve stump approaches revealing less MN death than NMJ‐dependent approaches in the same motor pool (Fogarty et al., 2018; Fogarty & Sieck, 2023).

## 5 CONCLUSIONS

Overall, we find that the preponderance of studies in spinal cord and brainstem MN loss, particularly the higher‐quality ones, support the assertion of age‐associated MN loss in humans and model species. As always, the devil is in the details, with many negative studies recapitulating fundamental sampling errors, and many positive studies in individual MN pools suffering from the pitfall of relying on NMJ integrity to evaluate MNs. As the neurogenic contributions to aging muscle decline become more heavily investigated, researchers must remain vigilant to ensure studies plagued by poor habits and design (see future directions) do not creep into the literature.

## AUTHOR CONTRIBUTIONS

**Sang Won Cheung:** Data curation; formal analysis; funding acquisition; investigation; methodology; validation; visualization. **Matthew J. Fogarty:** Conceptualization; data curation; formal analysis; funding acquisition; investigation; methodology; project administration; supervision; visualization.

## FUNDING INFORMATION

This study was supported by R01 AG086136 (MJF) from NIH.

## CONFLICT OF INTEREST STATEMENT

None of the authors has any conflicts of interest, real nor perceived, to disclose. The funders had no role in the preparation of this manuscript. For the manuscripts included within the scoping review, funders are disclosed within each article.

## ETHICS STATEMENT

This review article was conducted with the highest degree of attention to the details of the original studies. The authors did not perform any human or animal experiments in the production of this work.

## PATIENT CONSENT STATEMENT

No human subjects were used in the preparation of this work.

## Supporting information


**Data S1.** Preferred Reporting Items for Systematic reviews and Meta‐Analyses extension for Scoping Reviews (PRISMA‐ScR) Checklist.

## Data Availability

No original data was used in this study.
